# Dysregulated metal ion homeostasis underscores non-canonical function of CD8^+^ T cell during COVID-19

**DOI:** 10.3389/fmed.2023.1282390

**Published:** 2023-10-10

**Authors:** Kriti Khare, Partha Chattopadhyay, Priti Devi, Priyanka Mehta, Aakarshan Raina, Chinky Shiu Chen Liu, Kishore Tardalkar, Meghnad G. Joshi, Rajesh Pandey

**Affiliations:** ^1^Division of Immunology and Infectious Disease Biology, INtegrative GENomics of HOst-PathogEn (INGEN-HOPE) Laboratory, CSIR-Institute of Genomics and Integrative Biology (CSIR-IGIB), Delhi, India; ^2^Academy of Scientific and Innovative Research (AcSIR), Ghaziabad, India; ^3^D. Y. Patil Education Society, Institution Deemed to be University, Kolhapur, Maharashtra, India; ^4^Department of Stem Cells and Regenerative Medicine, D. Y. Patil Education Society, Deemed to be University, Kolhapur, Maharashtra, India

**Keywords:** single cell RNA-seq, COVID-19, T cell heterogeneity, non-canonical, metal ion

## Abstract

**Introduction:**

Several efforts have been made to describe the complexity of T cell heterogeneity during the COVID-19 disease; however, there remain gaps in our understanding in terms of the granularity within.

**Methods:**

For this attempt, we performed a single-cell transcriptomic analysis of 33 individuals (4 healthy, 16 COVID-19 positive patients, and 13 COVID-19 recovered individuals).

**Results:**

We found CD8^+^ T cell-biased lymphopenia in COVID-19 patients compared to healthy and recovered individuals. We also found an optimal Th1/Th2 ratio, indicating an effective immune response during COVID-19. Expansion of activated CD4^+^ T and NK T was detected in the COVID-19-positive individuals. Surprisingly, we found cellular and metal ion homeostasis pathways enriched in the COVID-19-positive individuals compared to the healthy and recovered in the CD8^+^ T cell populations (CD8^+^ TCM and CD8^+^ TEM) as well as activated CD4^+^ T cells.

**Discussion:**

In summary, the COVID-19-positive individuals exhibit a dynamic T cell mediated response. This response may have a possible association with the dysregulation of non-canonical pathways, including housekeeping functions in addition to the conventional antiviral immune response mediated by the T cell subpopulation. These findings considerably extend our insights into the heterogeneity of T cell response during and post-SARS-CoV-2 infection.

## Introduction

The cellular immune response is a major component of immune defense, where its role involves the recognition of pathogens and activation of the entire cascade of defense mechanisms. COVID-19 disease, caused by SARS-CoV-2, has become a benchmark for any pandemic that has happened so far due to a viral pathogen. From mild to critical, the range of clinical symptoms elicited by SARS-CoV-2 infection has made it one of the most widely studied viral diseases (1). T cell-mediated immunity is critical in providing protection against several infectious diseases, and therefore its role in COVID-19 disease is of utmost importance (2). Several studies have reported the T cell immune profile in COVID-19-positive individuals, where in particularly CD4^+^ and CD8^+^ T cells are observed to be reduced with disease progression (3, 4). This lymphopenia, with a decrease in the T cell immune population, has been associated with disease severity (5). Yet, other findings suggest that a poor clinical outcome is linked with the hyperactivation profile of T cells (6, 7). These two extreme cases, where both depletion and hyperactivation of T cells occur, lead to difficulty in understanding the optimal and successful T cell response induced upon SARS-CoV-2 infection. Whether the T cell response is helpful or harmful in COVID-19 is a matter of concern, while it is also relevant to discover the most affected T cell sub-populations and their heterogeneous responses. Therefore, it is necessary to delineate the granularity of T cell responses during SARS-CoV-2 infection to uncover the significance of cellular immunity in several other infectious diseases.

**Figure 1:**
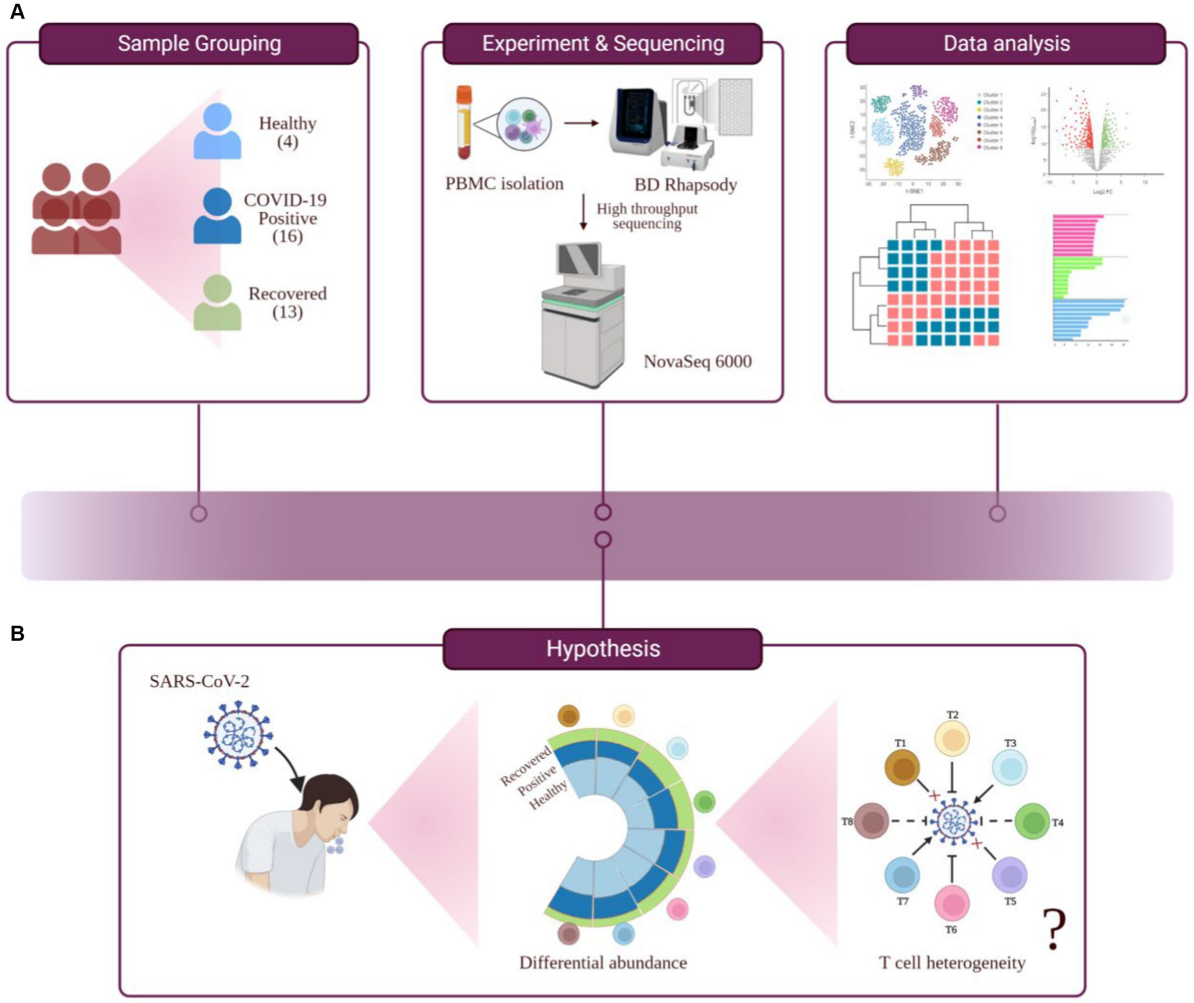
Study outline, workflow of experiment and hypothesis testing. **(A)** Study outline and experimental workflow, comprising sample collection and grouping, PBMC isolation, single-cell sequencing, data analysis, and interpretation. **(B)** Hypothesis toward investigating the functional role of T cell heterogeneity in SARS-CoV-2 infected individuals.

Lymphopenia, along with its modest immunological disruption, has been extensively studied as a characteristic of COVID-19 progression, although it has yet to be properly elucidated. During COVID-19, a decline in the T cell population leads to an insufficient and unbalanced immune response (8). Therefore, the purpose of this study was to determine if all T cell subsets behave similarly or whether a few are responsible for effective and efficient immune responses during COVID-19. Therefore, this study hypothesizes that T cell heterogeneity occurs in COVID-19-positive individuals, which could influence the severity of the disease or its remission (Figures 1A,B).

Here, we have investigated a heterogeneous T cell response in COVID-19 patients compared to healthy and recovered individuals. The experimental workflow is depicted in Figure 1A. We found a prominent CD8^+^ T cell-specific lymphopenia in infected individuals, in concordance with the previous findings. We also found activated CD4^+^ T and NK T (natural killer T) cell populations to be elevated, compared to other cell types, in COVID-19-positive individuals. We also report an optimal Th1/Th2 (T-helper) ratio in COVID-19 positive patients. Interestingly, we found metal ion homeostasis and cell-cycle regulation pathways, along with other immune-related pathways, altered in COVID-19 positive individuals. These pathways were particularly associated with CD8^+^ TCM (central memory T cell), CD8^+^ TEM (effector memory T cell), and activated CD4^+^ T. We found an association between metal ion homeostasis and immune response within the T cell population during COVID-19 which indicates the involvement of housekeeping functions in the regulation of T cell immune response.

## Results

### T cells heterogeneity in COVID-19-positive compared to healthy and recovered individuals

**Figure 2:**
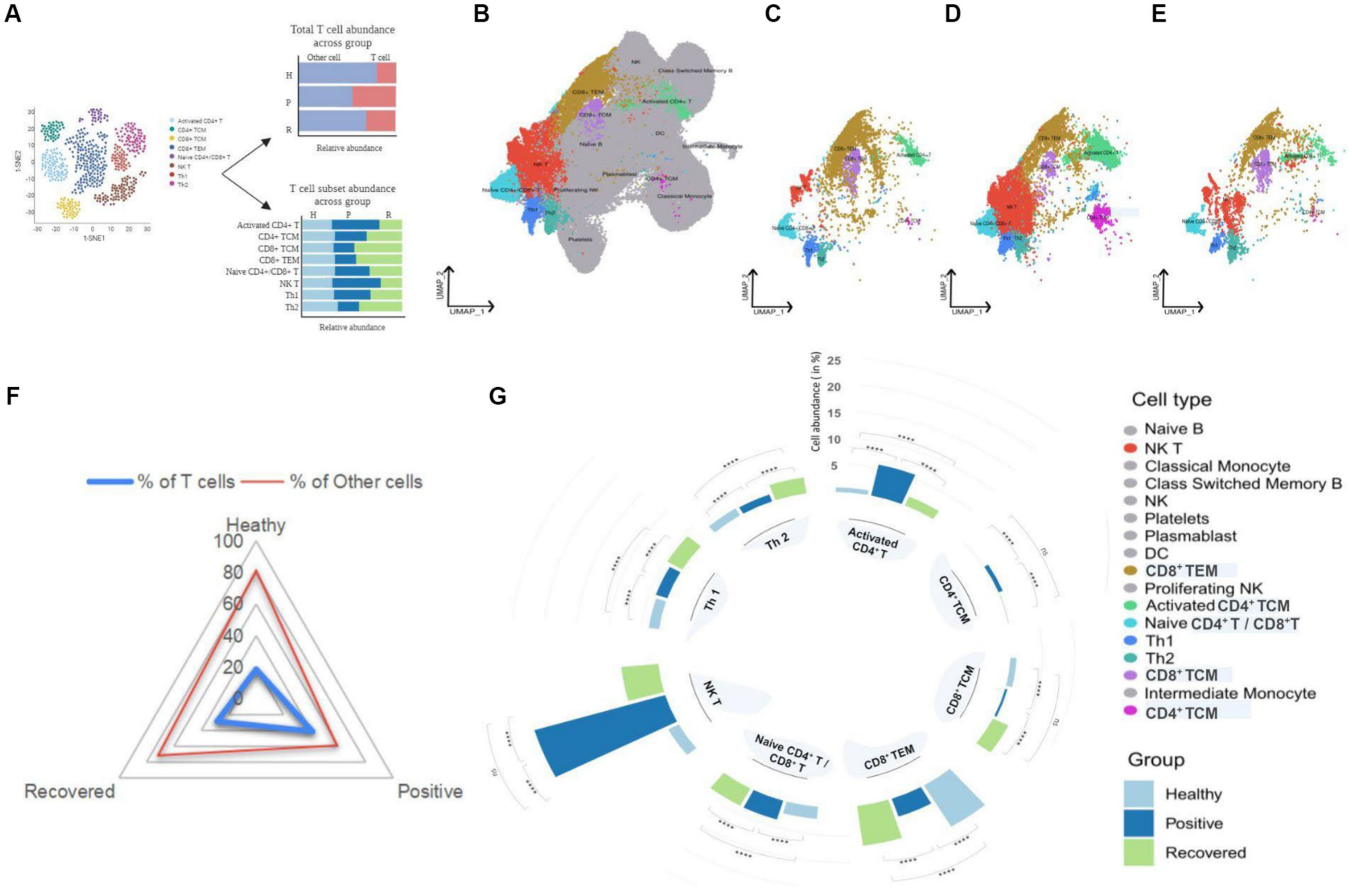
UMAP visualization of peripheral blood immune cells and distribution of T cell subsets in healthy, positive, and recovered individuals. **(A)** Graphical representation of the panel outline. **(B)** UMAPs of 124,476 single cells with 17 cell types annotated from single-cell sequencing of PBMCs from the individuals. **(C–E)** UMAPs of eight T cell subsets in healthy, COVID-19 positive and recovered individuals, respectively. T cell subsets are colored with their respective colors, while the rest of the cell types are gray in color. **(F)** Radial plot with T cell abundance compared to other cells in healthy, positive, and recovered individuals. **(G)** A circular bar chart showing the relative abundance of T cell subsets across healthy, positive, and recovered; significance calculated by the Fisher exact test (*p* > 0.05: ns, *p* < 0.05 > 0.01: *, *p* < 0.01 > 0.001: **, *p* < 0.001 > 0.0001: ***, *p* < 0.0001: ****).

Complex heterogeneity of cellular dynamics is clearly apparent in our cohorts, where we have identified a total of 17 cell types. These include B cell subsets, T cell subsets, NK cells, dendritic cells, monocytes, and platelets. Interestingly, almost 50% of the identified population were T cells, thereby suggesting a prominent involvement of T cell response during COVID-19 (Figure 2A). T cells form one of the primary components of the antiviral immune response. Their ability to recognize infected cells and eliminate them through intrinsic cytotoxicity or by supporting antibody production and subsequent neutralization is essential for an effective specific immune response against viruses and for the development of lasting immunity. With this understanding, we focused on eight T cell subsets of the total 17 cell types identified, which include activated CD4^+^ T, CD8^+^ TCM, CD8^+^ TEM, CD4^+^ TCM, naive CD4^+^/CD8^+^ T, NK T, Th1, and Th2, across healthy, COVID-19 positive, and recovered cohorts (Figures 2B–E; Supplementary Figures S1A,B).

We investigated the abundance of T cells in the healthy, COVID-19 positive, and recovered individuals and found significant differences across the groups. COVID-19 positive group was observed to have the highest proportion of T cells, followed by recovered and healthy individuals (Supplementary File 1; Figure 2F). However, when examined at the T cell subset level, it was not the same. The antiviral immune response mobilizes T cells, which recognize and control viral expansion. The increase in T cell numbers, especially effector, cytotoxic T cells and memory T cells, is a crucial part of this response to combat the infection and establish long-term immunity (9, 10). However, research has shown that this paradigm is lost during SARS-CoV-2 infection and vastly dysregulated T cell frequencies are observed across disease states (3, 4). We found five T cell populations, activated CD4^+^ T, naive CD4^+^/CD8^+^ T, CD4^+^ TCM, NK T, and Th1 cells, to be significantly increased in COVID-19 positive individuals. Of interest, we observed a significant reduction in CD8^+^ TCM, CD8^+^ TEM, and Th2 cells in the COVID-19-positive compared to healthy and recovered individuals. In particular, the Th1/Th2 ratio was balanced in a way such that the Th1 cells showed an increase while Th2 cells decreased in the COVID-19 patients when compared to the healthy individuals. Also, we observed a higher response of Th2 in the recovered individuals which may allude to the immunosuppressive and repair function of these subsets post-infection. Activated CD4^+^ T and NK T cells were the most abundant cell types compared with other cell types in the COVID-19 patients. The CD4^+^ T cell population was maintained within the COVID-19 positive individuals where naïve, activated, and central memory CD4^+^ T cell subsets showed a significant increase upon SARS-CoV-2 infection. Increased relative abundance of activated CD4^+^ T cells also generates more central memory CD4^+^ T cells which may favor better outcomes for infected individuals. An effective T cell response typically involves transitioning from a naïve state to an activated state that culminates in the formation of long-lasting memory. When stimulated by an antigen, naïve T cells become activated and undergo subsequent expansion (11). Subsequently, a subset of these activated cells undergoes maturation, giving rise to various memory subsets, including central memory T cells, effector memory T cells and tissue-resident memory T cells (12). The larger pool of activated CD4^+^ T cells presents more opportunities for some of them to undergo this differentiation into memory T cells which contribute to the development of augmented immune response during subsequent reinfections (13). However, it is crucial to recognize that this differentiation process is intricate and influenced by several factors, such as the duration and intensity of antigenic stimulation, the specific cytokine environment, and various transcriptional factors (14). Conversely, lack of memory CD8^+^ T cell formation in SARS-CoV-2 infected patients likely indicates dysregulated CD8^+^ T cell population during the disease course. Together, this depicts that CD4^+^ T cell function is maintained during COVID-19 which may benefit the infected patient toward recovery, while impaired CD8^+^ T cell population could have a plausible contribution toward disease progression and severity (Supplementary File 1, Figure 2G). For each relative abundance proportion, the difference in healthy vs. positive and positive vs. recovered was significant for all the T cell subsets. Altogether, this suggests that during COVID-19, only specific T cell subsets are affected while most of them might be involved in eliciting an active immune response in SARS-CoV-2 infected individuals for them to recover post-infection.

### Pattern of differentially expressed genes in COVID-19 positive compared to the healthy and recovered

**Figure 3:**
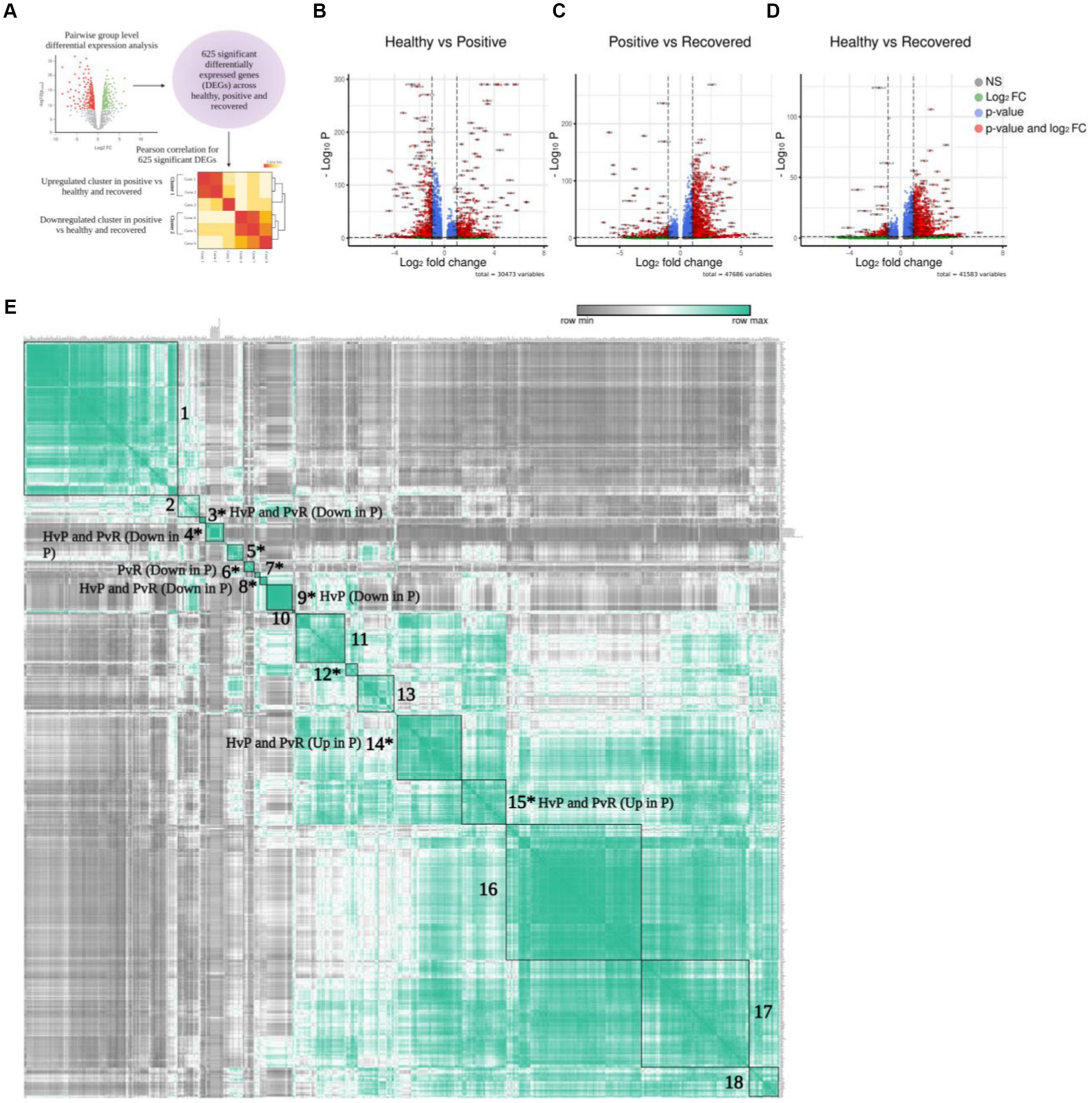
Differential gene expression of T cells. **(A)** Graphical representation of the panel outline. **(B–D)** Volcano plot depicting all the differentially expressed genes in clustered pairwise comparison (Healthy vs. Positive. Positive vs. Recovered, and Healthy vs. Recovered) across the T cell population. The green dots describe genes with log2 fold change more than ±1.5; the red dots represent significant genes based on both q-value and log2 fold change. **(E)** Pearson correlation plot showing the significant differentially expressed genes across the pairwise comparison groups. The boxes denote genes that are differentially expressed and have a positive correlation score that are grouped together. Asterisk marked clusters are found to be significant in the Pearson correlation analysis (*p*-value ≤ 0.05).

To further elucidate the above-mentioned discrepancies within the T cell subsets, we compared the transcriptomic profile via differential gene expression analysis with T cell subsets between healthy/positive, positive/recovered and healthy/recovered (Figure 3A), which has been depicted through a volcano plot (Figures 3B–D). Of all, we identified 285 significant differentially expressed genes (DEGs) for the COVID-19-positive/healthy (123 up and 162 down in the positive), 384 DEGs for COVID-19 positive /recovered (330 up and 54 down in the positive), and 162 DEGs for recovered/healthy (8 up and 154 down in the recovered; FDR ≤ 0.05 and |log2foldchange| ≥ 1.5; Supplementary File 2). The overlap between the upregulated and downregulated genes is due to the presence of a gene in more than one cell type within a particular group.

To characterize the correlation between DEGs, we conducted a Pearson correlation analysis to identify similarities and differences among the three comparison groups. This was done because genes frequently interact with one another rather than acting independently. In total, 625 unique and significantly differentially expressed genes found across healthy, COVID-19 positive, and recovered were taken for Pearson correlation and hierarchical clustering. The average expression of all these significant genes was used for the analysis (Supplementary File 3), which revealed 18 positively correlated clusters (Supplementary Files 4, 5). Out of 18 clusters, 10 were found to be statistically significant (*p* ≤ 0.05; Supplementary File 6; Figure 3E). Interestingly, we found that these clusters were expressed across a particular group. For example, clusters 14 and 15 represented significant DEGs that were upregulated in the COVID-19 positive in both the healthy/positive and positive/recovered comparison cohorts. Similarly, clusters 3, 4, and 8 showed downregulated DEGs expression in the COVID-19 positive in both above comparison cohorts. This suggests that the genes within a particular cluster might be performing a coordinated function due to their directional expression. On the other hand, clusters 6 and 9 uniquely expressed DEGs that were downregulated in the positive/recovered and healthy/positive, respectively. Cluster 5, 7, and 12 showed both upregulated and downregulated expression of genes in positive across healthy/positive and positive/recovered comparison cohorts. This indicates a directional and diverse with group specific expression of genes, thus pointing to their exclusive nature.

### Activated CD4^+^ T and CD8^+^ T cell population specific enriched pathways in the COVID-19 patients

A total of 172 unique DEGs across 10 significant clusters were filtered and divided into two broad categories for each comparison group based on the directions of differential expression, i.e., upregulated, or downregulated. In total, we had six groups, namely upregulated and downregulated DEGs of COVID-19 positive in the healthy/positive; and positive/recovered, along with upregulated and downregulated DEGs of recovered in the healthy/recovered (Supplementary File 7).

**Figure 4:**
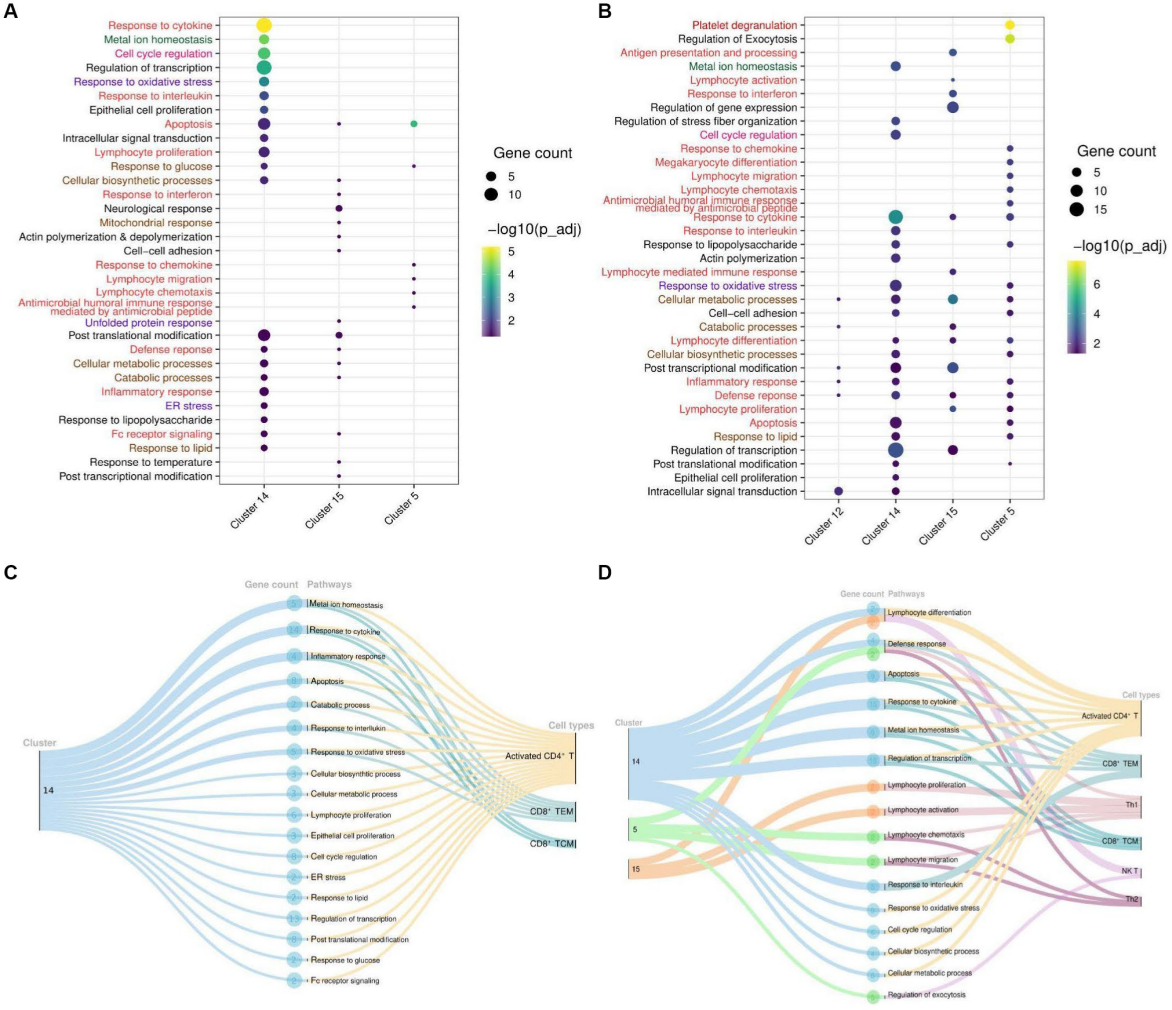
Cluster and T cell specific pathways in COVID-19-patients. **(A)** Dot Plot depicting upregulated DEGs and cluster specific enriched GO pathways in COVID-19 positive compared to healthy individuals. **(B)** Dot Plot depicting upregulated DEGs and cluster specific enriched GO pathways in the COVID-19 positive patients when compared with recovered individuals. **(C)** Sankey plots indicating cluster 14 derived significant pathways enriched in CD8^+^ TCM, CD8^+^ TEM, and activated CD4^+^ T cell types. **(D)** Sankey plots indicating clusters 14, 5, and 15 derived significant pathways enriched in CD8^+^ TEM, activated CD4^+^ T, Th1, CD8^+^ TCM, NK T, and Th2 cell types. Pathways are highlighted according to their broad categories; immune related are in red, homeostasis are in green, stress response are in purple; and metabolic response are in brown.

To delve deeper into the biological functions of genes, we selected all DEGs from significant clusters, and then only clusters containing significant pathways (up to 20 top pathways from each cluster) for further exploration. With upregulated and downregulated DEGs considered, we performed GO pathway analysis to discover the biological pathways enriched within a target cluster in the respective groups. Of all, we found both immune and non-immune related pathways enriched within the COVID-19-positive individuals compared to healthy and recovered individuals. Further, non-immune related pathways were broadly sorted into four categories; metabolism, homeostasis, stress response, and cell cycle regulation, as highlighted in Figure 4. The top 20 significant pathways for each cluster in particular group (Supplementary File 8) is depicted in Figures 4A,B. Notably, cluster 14 was found to be more enriched with upregulated DEGs associated biological pathways in the COVID-19 positive compared to the healthy individuals. Coincidentally, cluster 14 was also enriched with significant pathways in the COVID-19 positive compared to recovered individuals (Figure 4B). Pathways including inflammatory response, response to cytokine, lymphocyte differentiation, and proliferation were associated with upregulated DEGs, suggesting an activated immune response generated during COVID-19. Other pathways, including responses to cellular metabolic and biosynthetic processes, metal ion homeostasis, cell cycle regulation, and responses to glucose and lipids, were also enriched within COVID-19 positive patients. These pathways are essential to maintain the optimal cellular homeostasis that may aid in defining cell fate and associated defense response during SARS-CoV-2 infection. Also, dysregulation of these pathways during infection may benefit the viral replication and possibly lead to worsening of the disease (15–17). However, the upregulated DEGs in the healthy vs. recovered cohort did not reveal any relevant findings due to the presence of a single DEG. Similarly, the pathways enriched with downregulated DEGs for each comparison were only due to presence of one or two genes (redundant), therefore not taken forward (Supplementary Figures S1C–F).

To gain deeper and more granular insight into the biological pathways, we further decided to investigate the T cell subset associated with enriched pathways in each cohort. We found that most of the enriched pathways in COVID-19-positive individuals were derived from activated CD4^+^ T cells, followed by CD8^+^ TCM, and CD8^+^ TEM cells (Figures 4C,D). Essentially, pathways including response to cytokine, interferon, interleukin, and inflammatory were enriched in the upregulated DEGs (*NFKBIA, FOS, JUN, JUNB, TNFAIP3, ZFP36,* and *DUSP1*) associated with these T cell populations. Beyond this, other important pathways, including cell cycle metabolism (biosynthetic, catabolic, and cell cycle regulation pathways), response to glucose and lipids, oxidative stress (ER stress, unfolded protein response), and cellular homeostasis, were also found to be enriched in the upregulated DEGs (*PPP1R15A, RGCC, IRS2, HSP90AA1, MT2A, MT1E*, *MT1X, ATF4, RHOA, PPBP,* and *PF4*) within COVID-19 patients in activated CD4^+^ T cells compared to the healthy and recovered individuals, which indicate the response to a stimulus. These pathways are active or are regulated at the transcriptional level in the T cells in COVID-19 patients as compared to the healthy and recovered group. The enrichment of these non-canonical pathways in particular T cell subsets suggests that cellular homeostasis and stress responses like housekeeping functions could also be modulated vis-à-vis T cell during SARS-CoV-2 infection.

## Discussion

**Figure 5:**
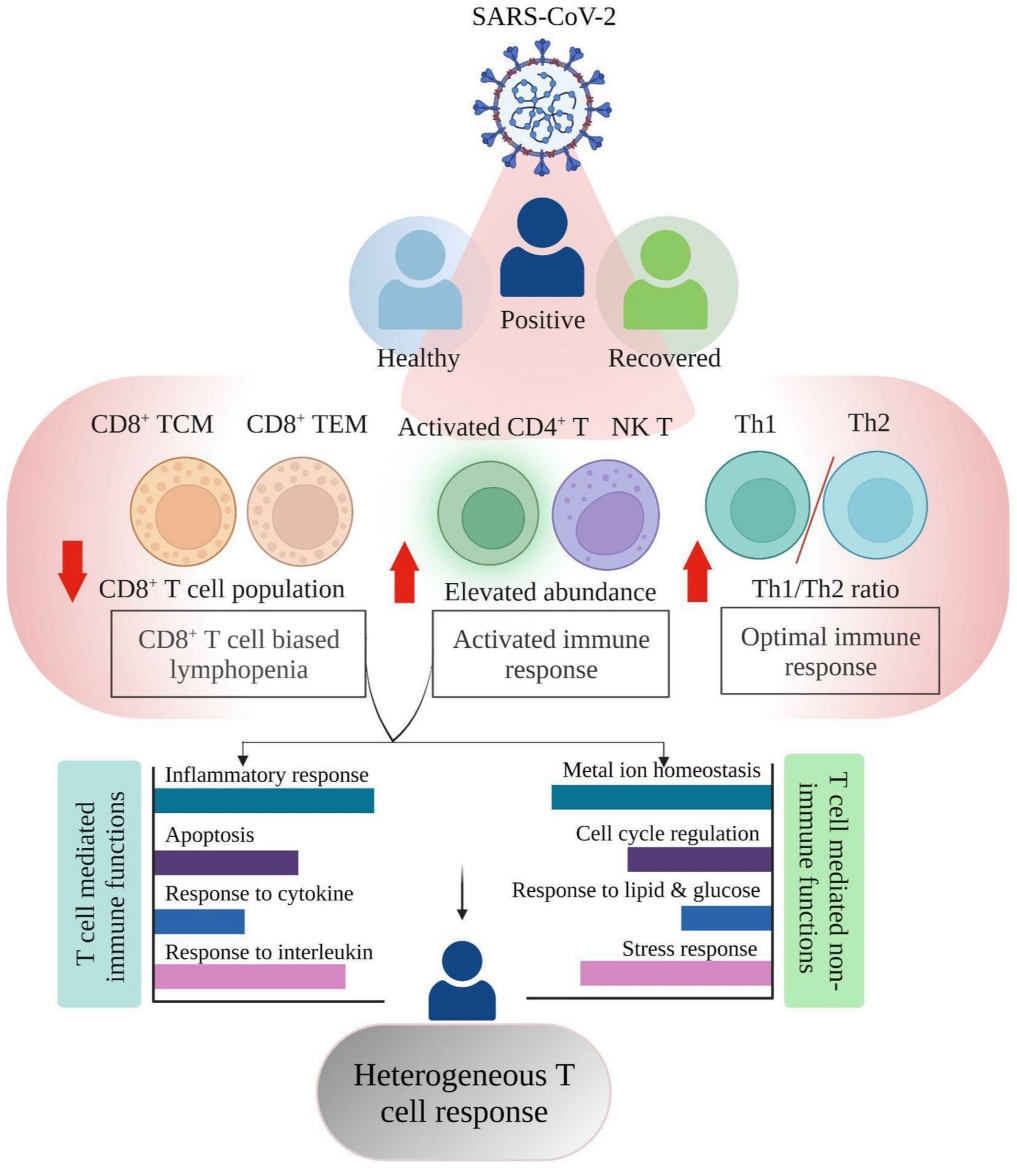
Summary highlighting the Single cell RNA-seq based T cell heterogeneous response in COVID-19 positive individuals.

The dysfunctional T cell response in COVID-19 has been widely studied since the beginning of the pandemic (18); however, there are major missing links and critical deficits in our understanding of the heterogeneity lying within the T cell mediated immune response. Our earlier study highlighted the activation of T cells in COVID-19 positive individuals along with dynamic antigen presentation and immune response. Employing previously generated single cell data, this study focused more in depth to explore the dynamics of T cell response in the COVID-19 positive compared to healthy and recovered individuals (Figure 5).

Despite the overall increase in T cell numbers in COVID-19 patients, the subsets did not follow a similar trend (Figures 2F,G). An increase in the relative abundance of activated CD4^+^ T, CD4^+^ TCM, and NK T cells was prominent, which may be considered as one of the factors for an active T cell response, however, the CD8^+^ T cell population (CD8^+^ TCM and CD8^+^ TEM) was reduced in COVID-19-positive individuals when compared with healthy and recovered individuals. Several studies have shown that both CD4^+^ T and CD8^+^ T cell decrease is associated with disease severity (19–22). However, in our dataset, only CD8^+^ T cells, not CD4^+^ T cells, were reduced. Thus, given the importance of CD8^+^ T cell population in virus control and its subsequent elimination (23), CD8^+^ T cell biased lymphopenia in SARS-CoV-2 infected individuals may lead to delayed viral clearance and further accelerate the disease progression, in concordance with previous findings (24, 25).

We also found an optimal Th1/Th2 ratio in the COVID-19 patients, where Th1 cells increased during SARS-CoV-2 infection while Th2 decreased (Figure 2G). A study shows that an efficient Th1-type immune response, after a viral infection, can eradicate it (26). The cytokine storm that results from an overactive immune response and increased cytokine production, however, leads to a Th2-type response and a poor prognosis for COVID-19 (27, 28). With disease severity, the Th1/Th2 ratio decreased (29) however in this study we found an increased Th1/Th2 ratio which implies that the T-helper response produced in these COVID-19 positive patients was optimum, which may have prevented the disease’s severity from worsening. Another observation was an expansion of activated CD4^+^ T and NK T cells in the COVID-19 positive individuals that was further reduced in recovered individuals. During severe COVID-19, there is a decline in NK T cell population where the cytotoxic activity of NK T cells becomes limited (30). Increased NK T cells however offer cytotoxicity toward SARS-CoV-2 infection which may aid in the recovery process (31). This may suggest an activated T cell response contributed by these cell subsets toward maintaining an optimal T cell response. From the above findings, it can be concluded that not all T cells are affected by COVID-19 while those that are engaged in eliciting an antiviral effect are majorly decreased, thus explaining the functional importance of the heterogeneous spectrum of T cell response during SARS-CoV-2 infection.

The transcriptomic profile and GO pathway analysis revealed relevant DEGs and their associated immune and non-immune related pathways in the COVID-19 positive individuals. Most of the immune related pathways enriched in the COVID-19 positive individuals were driven by upregulation of *NFKBIA, FOS, JUN, JUNB, TNFAIP3, ZFP36, DUSP1,* and *PPBP*. These genes were present among the prominent pathways, including cytokine, chemokine, interleukin, inflammatory response, and apoptosis (Figures 4A,B). Previous studies show that during COVID-19, the activation of cellular immune cascade highly depends upon release of interleukins and interferon (32), which may lead to efficient T cell mediated immune response (33), which is reflected through our study as well.

Also, irrespective of the healthy and recovered groups, a few genes, including *MT2A, MT1E*, and *MT1X* were involved in non-immune related pathways such as metal ion homeostasis. In our data, the response to cytokine pathway also shows upregulated expression of *MT2A* and *MT1X* in the COVID-19 patients. Of several factors including antioxidants, and heavy metals, the cytokine release is also known to trigger metallothionein expression which can lead to immunomodulatory results (34). This suggests that cellular homeostasis pathways might be altered via the induction of cytokine response during COVID-19. Previous studies have observed an increased expression of NF-kB pathway genes (*JUN*/*FOS*/*NFKBIA*) in the SARS-CoV-2 infected individuals, thereby activating the immune cascade (35). Thus, our data related to this finding since these genes were highly expressed in COVID-19 positive individuals compared to both healthy and recovered individuals.

Within the COVID-19 positive individuals, the distribution of relevant pathways was not across every T cell subpopulation. In both comparison groups (healthy/positive and positive/recovered), we found that activated CD4^+^ T and CD8^+^ T cell populations (CD8^+^ TCM and CD8^+^ TEM) majorly expressed enriched pathways in the COVID-19 patients (Figures 4C,D). Generally, T cell activation depends upon the cell cycle regulators (36) which aid in the proliferation and subsequently increased production of effector cytokine to challenge the invading pathogen (37). In concordance, we found cell cycle regulation, lymphocyte differentiation, and proliferation enriched in the activated CD4^+^ T cells, possibly contributing to their activation in the COVID-19 positive individuals.

Metal ions play essential roles in both host and microbial metabolism, being integral to key enzymatic activities involved in processes like DNA synthesis and cellular respiration. Their availability influences host immune responses and microbial defenses during infections, leading to efforts by the host cells to regulate metal access, impacting cellular response and outcomes during infection. Microbes activate specific pathways to ensure a steady supply of metals crucial for their pathogenicity and immune evasion (38). Impact of metal ions on immune response has been previously studied where metal ions, including lead, mercury, and cadmium, can disrupt T cell function, leading to dysregulation through changes in cytokine production, proliferation, and signaling pathways (39). Zinc is considered essential for T cell activation and proliferation during viral infections, but imbalances can hinder T cell function (40). Iron’s role in cellular processes affects immune cell function, including T cell responses (41). Metal ions also act as cofactors in immune-regulating metalloenzymes, impacting T cell function during viral infections (42). Moreover, metallothionein expression is regulated by innate and adaptive immune cells in response to pathogens, cytokines, and stress, contributing to metal-mediated immune modulation (43). Thus, during a viral infection, there have been reports of T cell dysregulation and its correlation with metal ion homeostasis (44–47), however their association with heterogeneous T cell response during viral infection has not been thoroughly explored. Interestingly, we found metal ion homeostasis pathway associated with metallothionein (*MT2A, MT1E*, and *MT1X*) genes enriched within CD8^+^ TCM, CD8^+^ TEM, and activated CD4^+^ T cells in the COVID-19 positive individuals (Figures 4C,D). Having decreased frequency of CD8^+^ T cell subsets together with increased expression of both proinflammatory cytokine response genes and metallothionein could allude to the impaired profile of CD8^+^ T cell population upon SARS-CoV-2 infection. An association between T cell dysfunction and *MT1* and *MT2* gene expression has been observed in LCMV viral infection (48). A previous study also implies that high expression of metallothionein genes may lead to CD8^+^ T cell dysfunction where targeted deletions of these metal ions may revert the effect (49). Taking this into account, our data may suggest a dysregulated T cell-mediated cellular homeostasis during COVID-19. It is reported that virus infection increases the ER stress which further enhances unfolded protein response (UPR) to neutralize the detrimental effect and restore ER homeostasis which is also evident in COVID-19 patients in activated CD4^+^ T cells (50). In concordance, we also found an enriched ER stress response in activated CD4^+^ T cells in COVID-19 positive individuals. Also, apoptosis pathways enrichment in the COVID-19 patients mainly in CD8^+^ TEM, CD8^+^ TCM, and activated CD4^+^ T cells reminds us of lymphopenia, which reduces the number of T cells compared to healthy and recovered individuals. So, enrichment of ER stress and apoptosis pathways together in activated CD4^+^ T and CD8^+^ T cell populations of COVID-19 patients’ points toward the hyperactivated and dysregulated T cell specific response during the disease phase. Altogether, there is a heterogeneous and dynamic T cell-mediated response during COVID-19 where majority of the T cell subsets are playing beneficiary role while specific ones are affected probably via dysregulation of homeostatic and housekeeping functions.

## Conclusion

In this study, we have highlighted the T cell heterogeneity in COVID-19 positive individuals compared to healthy and recovered individuals where lymphopenia is biased toward CD8^+^ T cell population. An optimal ratio of Th1/Th2 in SARS-CoV-2 infected individuals provides active T cell mediated immune response. Notably, we observed activated CD4^+^ T, CD8^+^ TCM, and CD8^+^ TEM cells mediated regulation of cellular and metal ion homeostasis in addition to their primary immune functions. This suggests the importance of T cell mediated response both immune and non-immune specific that may reflect different layers of alterations in COVID-19 patients and needs to be investigated in future toward understanding the T cell heterogeneity in other infectious diseases as well.

## Materials and methods

### Sample collection, processing, and library preparation

Samples were collected at Dr. D. Y. Patil Medical College, Hospital, and Research Institute, Kolhapur, Maharashtra, India. In total, 33 individuals were recruited for this investigation, consisting of 4 healthy, 16 COVID-19 positive, and 13 recovered individuals (4 weeks post-qRT-PCR negative result). The clinical metadata for these individuals is available in Supplementary File 9. According to the manufacturer’s protocol, the PBMCs were isolated from 5 mL blood using a BD Vacutainer® CPT™ Cell Preparation Tube. According to earlier research (31), a single-cell Whole Transcriptome Analysis (WTA) library was generated using the BD Rhapsody™ Whole Transcriptome Analysis (WTA) Amplification Kit and the BD™ Single-Cell Multiplexing Kit-Human where 2 lakh cells per sample were collected and labeled. For single cell capture, every cartridge on the BD Rhapsody express single-cell analysis system was loaded with 30,000 pooled cells, and cDNA synthesis was performed, as per the manufacturer’s instructions (Doc ID: 210967 Rev. 1.0). Single-cell libraries were sequenced on the NovaSeq 6000 platform using the S2 reagent kit at 30,000 reads/cell and with paired-end 101 cycles. The sequencing of these individuals yielded 163,197 cells, of which 124,726 were retained after low-quality cell removal. These cells were then subjected to downstream analysis, which included batch effect removal, normalization, dimensional reduction, unsupervised clustering, cell type identification, and, finally, differential expression analysis.

### scRNA-seq data processing, clustering, and cell-type annotation

The sequencing data were processed and analyzed using the BD Rhapsody WTA analysis pipeline, according to the manufacturer’s guidelines (Doc ID: 47383 Rev. 9.0). For further analysis and visualization, the count matrix with repeated substitution error correction was taken into the Seurat R package (51). In total, 163,197 cells were detected. Using SCTransform V2 (52, 53), the low-quality cells were removed, followed by batch effect removal and normalization. Unsupervised clustering was performed and cells were visualized at 0.4 resolution using UMAP. Finally, clusters were annotated both manually (CellMarkerDB and PanglaoDB) (54, 55) and via automated methods (Azimuth and scPred) (52, 56).

### Differential gene expression for pairwise group in T cells

Using the Seurat FindMarker function, differential gene expression analysis was conducted based on clustered paired data (healthy vs. COVID-19, COVID-19 vs. recovered and healthy vs. recovered). Log2fc 1.5 and p_adj ≤ 0.05 were used as cutoffs for filtering differentially expressed genes. Differential gene expression analysis was done T cell cluster-wise using the r package DESeq2 (57) across the three groups. All three comparison groups are plotted as volcano plots using the R package EnhancedVolcano (58).

### Pearson correlation analysis

To understand the relationship between comparison groups, Pearson correlation with hierarchical clustering was performed with the statistically significant differentially expressed genes with the rcorr () function in the Hmisc R package (59) and represented as a heatmap.

### Gene ontology pathway analysis

For significant clusters, gene ontology biological pathway analysis was performed using the Enrichr database (60). A cutoff was applied (p_adj ≤ 0.05) for selecting significant pathways and R package ggplot2 was used to visualize them (61).

## Data availability statement

The data presented in the study are deposited in the NCBI GEO repository, accession number GSE201088.

## Ethics statement

The studies involving humans were approved by the CSIR-IGIB Human Ethics Committee Clearance (Ref No: CSIR-IGIB/IHEC/2020-21/01) reviewed and authorized the studies involving human subjects. The studies were conducted in accordance with the local legislation and institutional requirements. The participants provided their written informed consent to participate in this study.

## Author contributions

KK: Data curation, Investigation, Visualization, Writing – original draft. PC: Data curation, Formal Analysis, Investigation, Visualization, Writing – original draft. PD: Data curation, Writing – original draft. PM: Data curation, Writing – review & editing. AR: Data curation, Writing – review & editing. CL: Writing – review & editing. KT: Resources, Writing – review & editing. MJ: Resources, Writing – review & editing. RP: Conceptualization, Funding acquisition, Methodology, Supervision, Writing – review & editing.

## Supplementary material

### SUPPLEMENTARY FIGURE S1

Cluster and T cell specific pathways in healthy, COVID-19-positive and recovered individuals.

### SUPPLEMENTARY FIGURE S2

Barplot with samples wise (severe vs mild) T cell heterogeneity among among T cell subsets across healthy, COVID-19 positive and recovered individuals.
