# Supplementary figures and images for: Dysregulated metal ion homeostasis underscores non-canonical function of CD8+ T cell during COVID-19
Source: Front Med (Lausanne). 2023 Oct 10;10:1282390. doi: 10.3389/fmed.2023.1282390 (PMC10598344; doi:10.3389/fmed.2023.1282390)

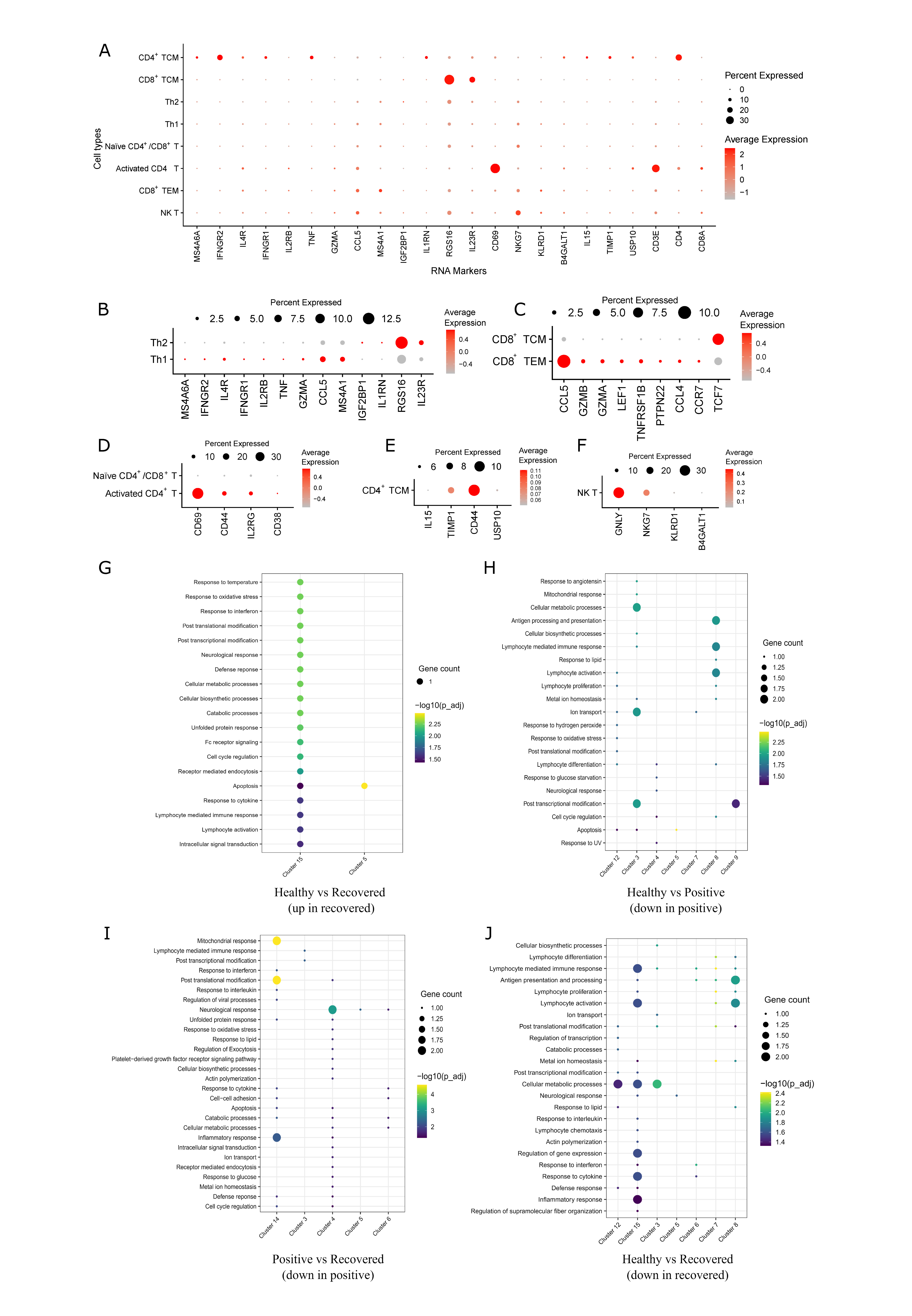

Supplement: Supplementary file 1 [file Data_Sheet_1.ZIP › supplementary figure 1.jpeg]

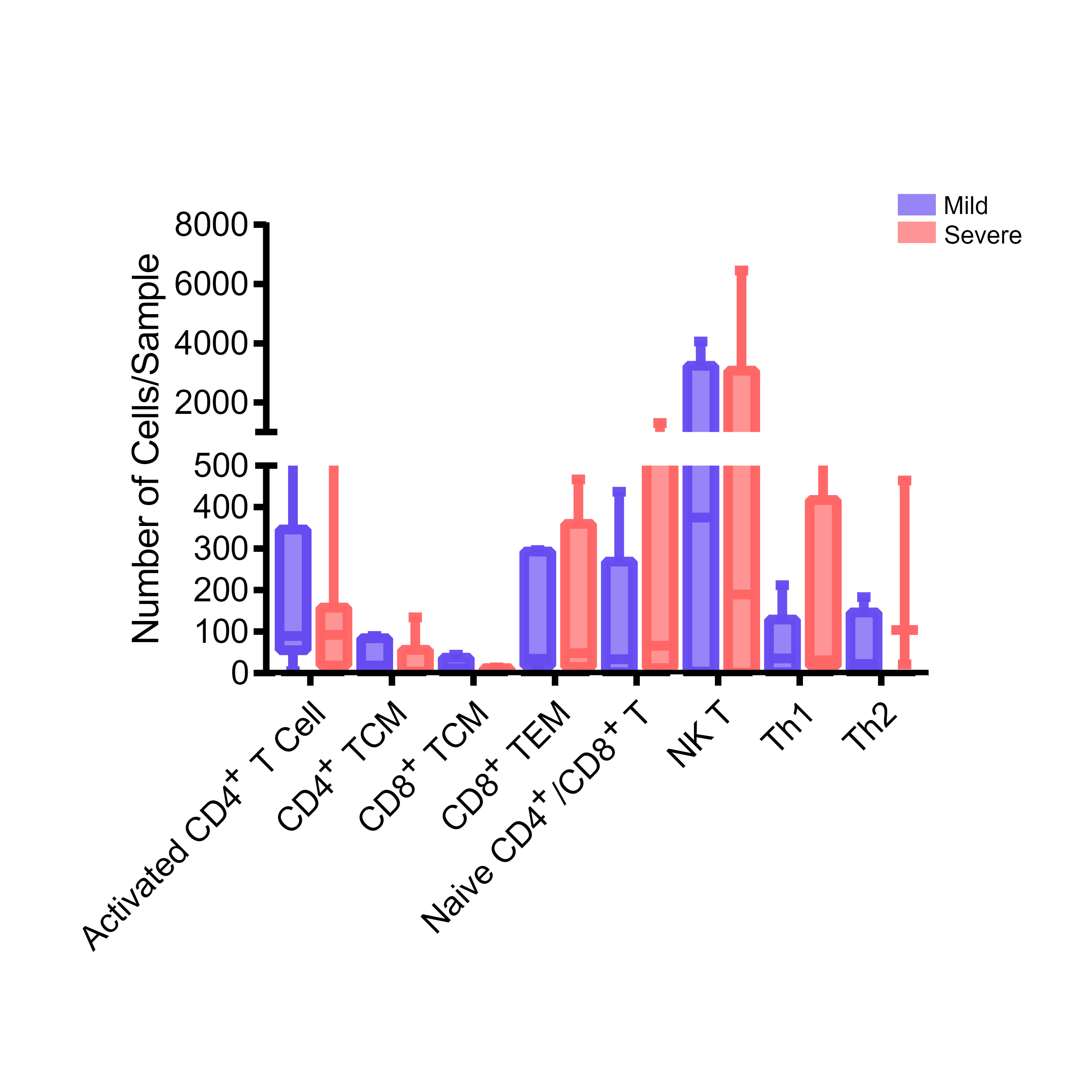

Supplement: Supplementary file 1 [file Data_Sheet_1.ZIP › supplementary figure 2.jpg]
